# High resolution imaging of the mitral valve in the natural state with 7 Tesla MRI

**DOI:** 10.1371/journal.pone.0184042

**Published:** 2017-08-30

**Authors:** Sam E. Stephens, Serguei Liachenko, Neil B. Ingels, Jonathan F. Wenk, Morten O. Jensen

**Affiliations:** 1 Department of Mechanical Engineering, University of Arkansas, Fayetteville, Arkansas, United States of America; 2 Department of Biomedical Engineering, University of Arkansas, Fayetteville, Arkansas, United States of America; 3 Division of Neurotoxicology, National Center for Toxicological Research, US Food and Drug Administration, Jefferson, Arkansas, United States of America; 4 Department of Mechanical Engineering, University of Kentucky, Lexington, Kentucky, United States of America; Worcester Polytechnic Institute, UNITED STATES

## Abstract

Imaging techniques of the mitral valve have improved tremendously during the last decade, but challenges persist. The delicate changes in annulus shape and papillary muscle position throughout the cardiac cycle have significant impact on the stress distribution in the leaflets and chords, thus preservation of anatomically accurate positioning is critical. The aim of this study was to develop an *in vitro* method and apparatus for obtaining high-resolution 3D MRI images of porcine mitral valves in both the diastolic and systolic configurations with physiologically appropriate annular shape, papillary muscle positions and orientations, specific to the heart from which the valve was harvested. Positioning and mounting was achieved through novel, customized mounting hardware consisting of papillary muscle and annulus holders with geometries determined via pre-mortem ultrasonic intra-valve measurements. A semi-automatic process was developed and employed to tailor Computer Aided Design models of the holders used to mount the valve. All valve mounting hardware was 3D printed using a stereolithographic printer, and the material of all fasteners used were brass for MRI compatibility. The mounted valves were placed within a clear acrylic case, capable of holding a zero-pressure and pressurized liquid bath of a MRI-compatible fluid. Obtaining images from the valve submerged in liquid fluid mimics the natural environment surrounding the valve, avoiding artefacts due to tissue surface tension mismatch and gravitational impact on tissue shape when not neutrally buoyant. Fluid pressure was supplied by reservoirs held at differing elevations and monitored and controlled to within ±1mmHg to ensure that the valves remained steady. The valves were scanned in a 7 Tesla MRI system providing a voxel resolution of at least 80μm. The systematic approach produced 3D datasets of high quality which, when combined with physiologically accurate positioning by the apparatus, can serve as an important input for validated computational models.

## Introduction

While in vitro imaging of the mitral valve (MV) has advanced tremendously over the last decade [1–3], a number of challenges still need to be addressed. The positioning and orientation of each papillary muscle (PM) and annular geometry are vital to proper function. It has been shown that leaflet strain and the chordal force balance are significantly impacted by the physiologically appropriate shape of the annulus [4–6] and PM position [7–9]. In addition to the overall position of the PMs, the orientation of the muscle tip with respect to the annulus can also have a similar effect since the chordae tendineae (CT) do not all insert into the PMs at one single, common point. Rather, the CT have a spatial distribution whose position with respect to the annulus varies with tip angle. Since there is substantial variation between individual MVs, controlling PM position/orientation and annular shape to the specific native conditions for the individual valve will maintain native leaflet strain, proper coaptation, and physiologically correct force distribution in the CT. The importance of CT distribution [10] is included in current computational models [11], but the location and orientation of the PMs as well as the 3-dimensional annular shape in relation to PM position in high resolution imaging datasets do not reflect the original *in vivo* conditions. Pre-mortem measurements are required to correlate the *in vitro* conditions to that of the native valve in order to validate computational models against realistic geometries. Additionally, depending upon the medium in which the valve is imaged, the tissue may also be subject to gravitational forces which distort the shape, most significantly in the open valve and zero-pressure configurations. The use of a liquid fluid environment keeps the tissue in a natural state and neutrally buoyant configuration. The liquid also eliminates the risk of tissue shrinkage and CT sticking together as part of altering the surface tension on the leaflets and CT, which has been demonstrated to cause significant morphological changes of the tissue [12]. One attempt to resolve this issue is by fixation of the tissue with glutaraldehyde [2, 3], which by itself also cause tissue shrinkage [12].

The following is a description of an *in vitro* left heart apparatus and process by which high-resolution 3-dimensional images of the MV in the zero-load natural diastolic state as well as the pressurized systolic state can be obtained within a fluid environment utilizing anatomically correct precise PM positioning and orientation as well as a true-shaped annulus holder. The process is designed such that the apparatus reflects *in vivo* conditions within a particular animal by employing simple measurements from pre-mortem 2D or 3D ultrasound imaging.

## Methods

The imaging apparatus consists of an annulus clamp and PM holder to mount the valve, a case to support the mounting hardware and contain the fluid bath, and a pressure regulation system to ensure constant trans-mitral pressure throughout the imaging process. The individual parts of this system are described below.

The portions of the apparatus that are exposed to magnetic fields from the MRI are constructed from acrylic, 3D printed plastic, and brass for MRI compatibility. The case and fluid regulation system are reusable and can accommodate a range of valve sizes corresponding approximately to a 26–36mm commissure-commissure width. The annulus clamp and PM holder are customized and 3D printed to conform to the annular shape and PM positions/orientation of the individual valve of study. Customizations are made using pre-mortem ultrasound measurements of the MV that are then used to modify the mounting hardware designs through computer aided design software. Depending on the ultrasound equipment available (2D or 3D), different methods of constructing the geometries are deployed. For this initial study, the following dimensions were used: commissure to commissure, septal to lateral, and mean commissure to PM length [13].

**Fig 1:**
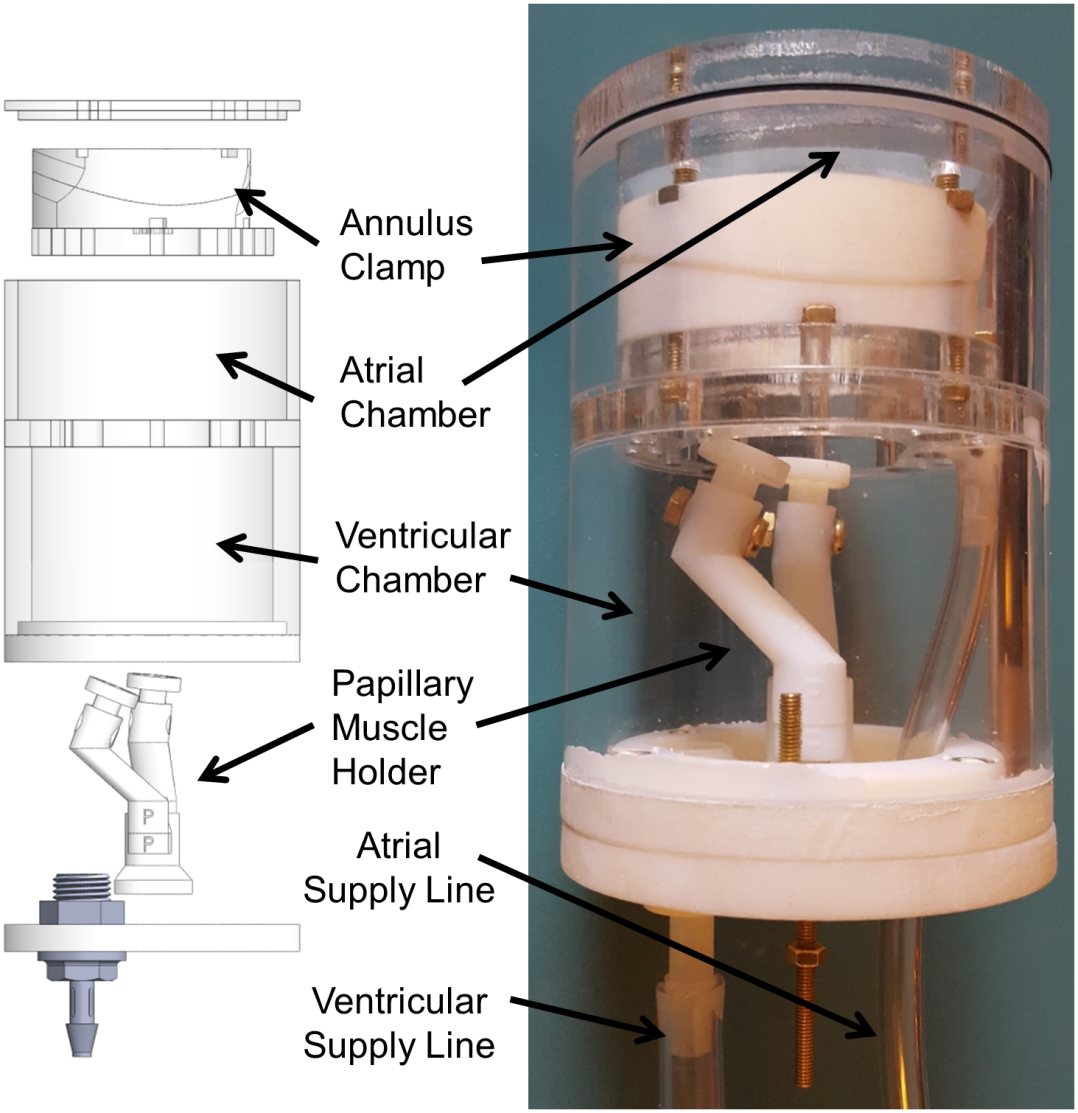
Simplified exploded view of apparatus (left) and assembled apparatus (right).

**Fig 2:**
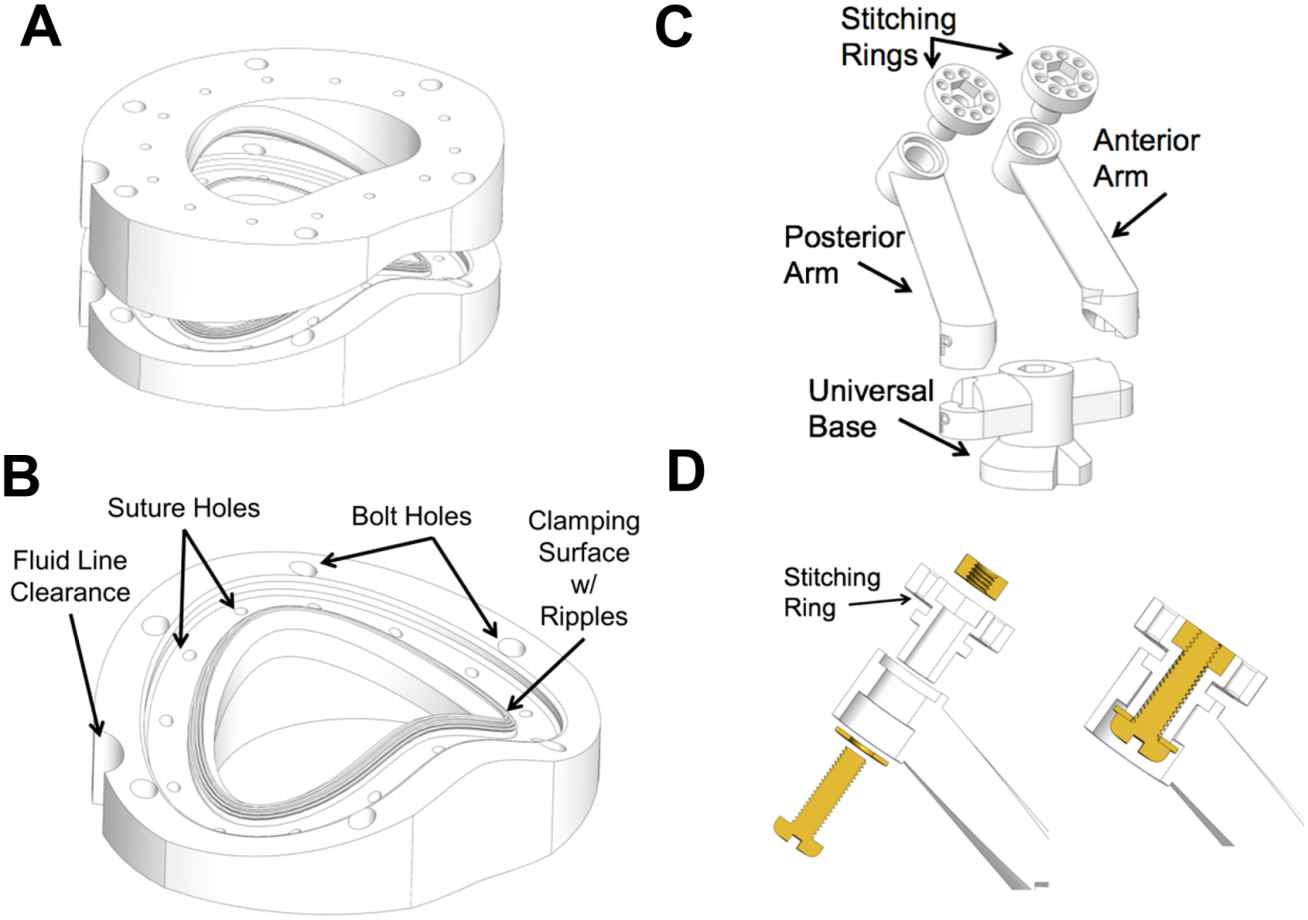
A) Annulus clamp B) Bottom of annulus clamp showing significant features C) Papillary muscle holder assembly D) Mounting of rotating stitching rings in section view.

Pressure across the valve is monitored and kept constant by a separate fluid controls system. The apparatus, excluding the control system, is shown in Fig 1 with major components labeled. Fig 2 shows the annulus clamp and PM holder with significant features labeled.

### Requirements for pre-mortem ultrasound measurements

**Fig 3:**
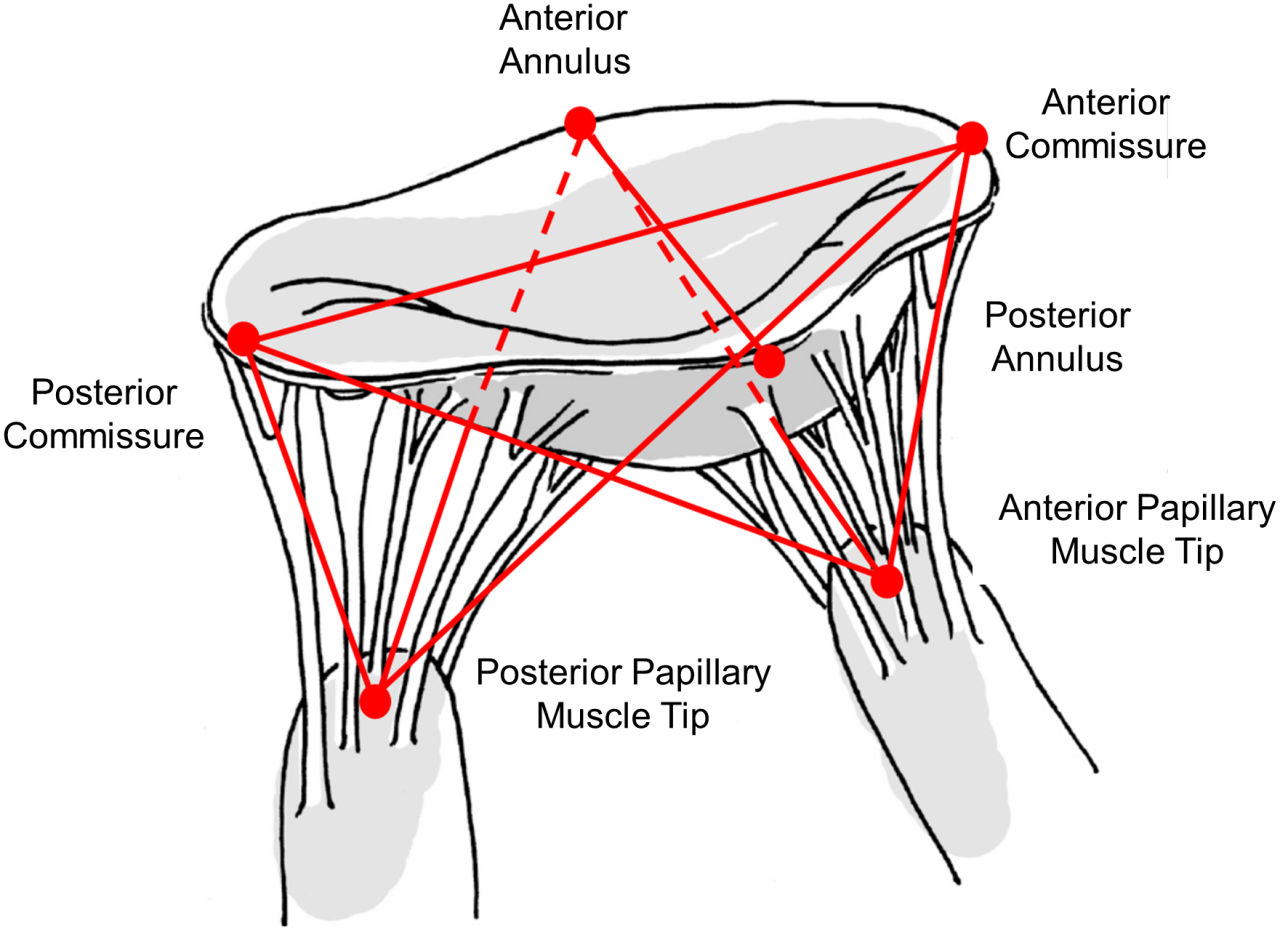
Minimum required mitral valve measurements for hardware customization. Red dots denote the ultrasound identifiable points, and the lines denote the simple 3D distance between those points.

2D or 3D echocardiogram measurements using Trans-esophageal echocardiography (TEE) for optimal signal quality can be used in a porcine animal model to set the dimensions of the valve mounting hardware. In the case of 2D data being available, the dimensions required to define the geometries of the annulus clamp are the commissure-commissure width (CC), the septal to lateral distance (SL), and the annular height (AH). The required points all correspond to anatomical features of the valve identifiable with ultrasound, which makes their identification and the measurement straightforward. Measurements required for modification of the PM holders are the distances from the anterior commissure (ACOM) to each PM tip, the posterior commissure (PCOM) to each PM tip, and the mid-anterior annulus (AA) to each PM tip. The minimum required measurements are illustrated in Fig 3 [5, 14]. While no other measurements are required by the algorithm employed in the case of 2D data being available, the accuracy of the annular shape will be improved by the addition of any other possible measurements, for example from 3D echo, as this will add more points to the curve fit performed in generating the annular shape. Additional points could include the trigone-trigone distance, height of saddle horn, and others. The experimental measurements for this study used porcine hearts due to the similarity with human hearts. All hearts that were used for testing purposes were obtained from a local abattoir.

### Mounting hardware customization

**Fig 4:**
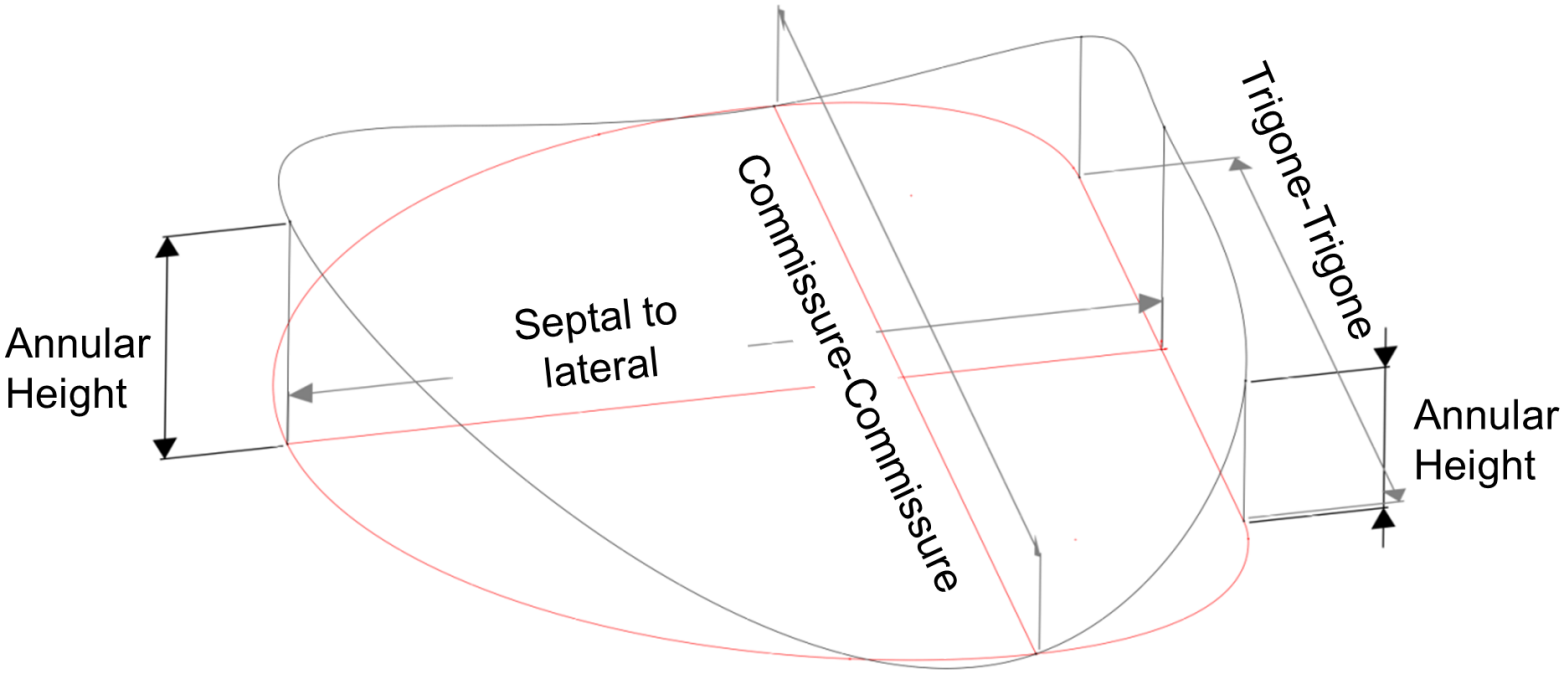
Adjustment points for annulus clamp customization.

The annulus clamp and PM holders are designed and edited using Solidworks 2016 (Dassault Systemes, Vélizy-Villacoublay, France). For the systolic dataset, the explanted valve will be MRI scanned in a closed configuration to prepare for future biomechanical measurement experiments; therefore, all *in vivo* dimensions are measured at a dedicated time point in the cardiac cycle, which can be at peak systolic left ventricular pressure or mid systole, as defined by the mid-point between dP/dt_max_ and dP/dt_min_ [6, 15, 16]. Mid diastole is then defined by the mid-point between dP/dt_min_ and dP/dt_max_. Hardware design begins by identifying the customization points of the annulus clamp which are illustrated in Fig 4. Reference points representing the projection of the commissures, trigones, and mid-anterior annulus are placed within a plane and dimensioned according to the CC and SL measurements. These points and the spline curve connecting them to form the annular projection are shown in red in Fig 4. Additional reference points, as available, representing the three-dimensional positions of the trigones and mid-anterior annulus are placed vertically from their corresponding projections and dimensioned using AH. The trigone-trigone width and the trigone height serve to establish the amount of 3D-shape variation present in the annulus. The points representing the three-dimensional mid-anterior annulus, trigones, and commissures are connected using a spline curve to create an annular profile. To customize the design to a new valve, CC, SL, and AH are changed to reflect new values. Any additional points measured can be readily added to this curve to improve the fit. The cross section of the clamp is drawn separately and is swept around this profile to generate a three-dimensional solid model. The midline of the cross section is maintained at a constant elevation angle throughout the circumference. All bolt holes and other features are automatically generated following adjustment of the annular dimensions.

**Fig 5:**
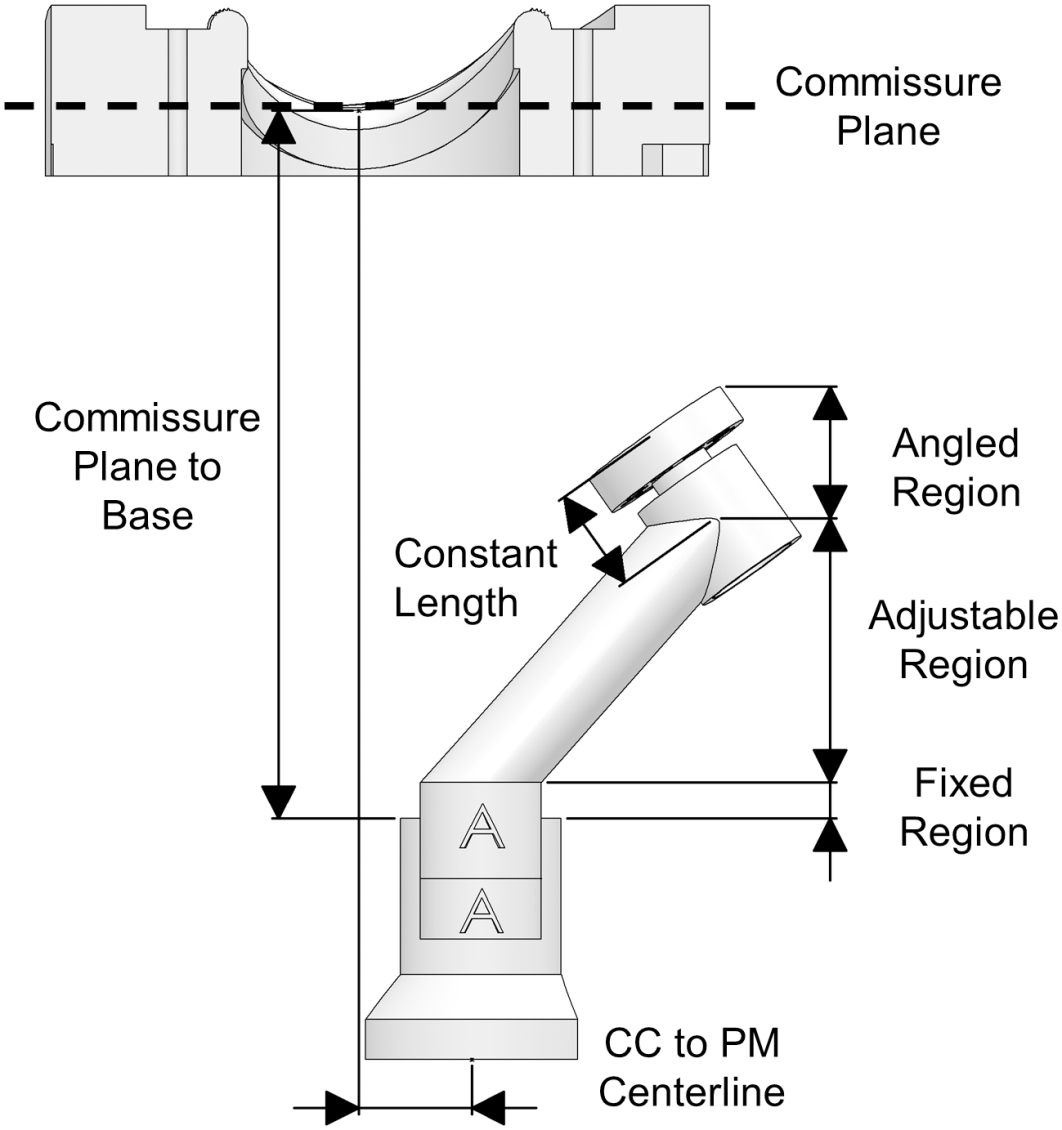
Papillary muscle holder sections and positioning with respect to the annulus clamp. PM: Papillary Muscle.

The PM holder is composed of five parts; a universal base, two universal stitching rings, and two customizable arms. The arms are responsible for allowing the PM positions and orientations to be specific to the animal from which the valve is harvested. There is a separate arm for each of the anterior and posterior PM. Each arm is composed of three regions; a fixed region, an adjustable region, and an angled region. The different regions are illustrated in Fig 5 along with the position of the PM holder with respect to the annulus clamp. The PM stitching rings are able to rotate in order for the PMs to align correctly with the annulus. The fixed regions do not change, and attach to the base. The adjustable region represents the middle of the arms and is responsible for controlling the spatial positioning of the PMs. The apical distance from the PMs to the annular plane is controlled by altering the height of this portion, while the position normal to the plane is controlled by offsetting the arm in the other two dimensions independently.

**Fig 6:**
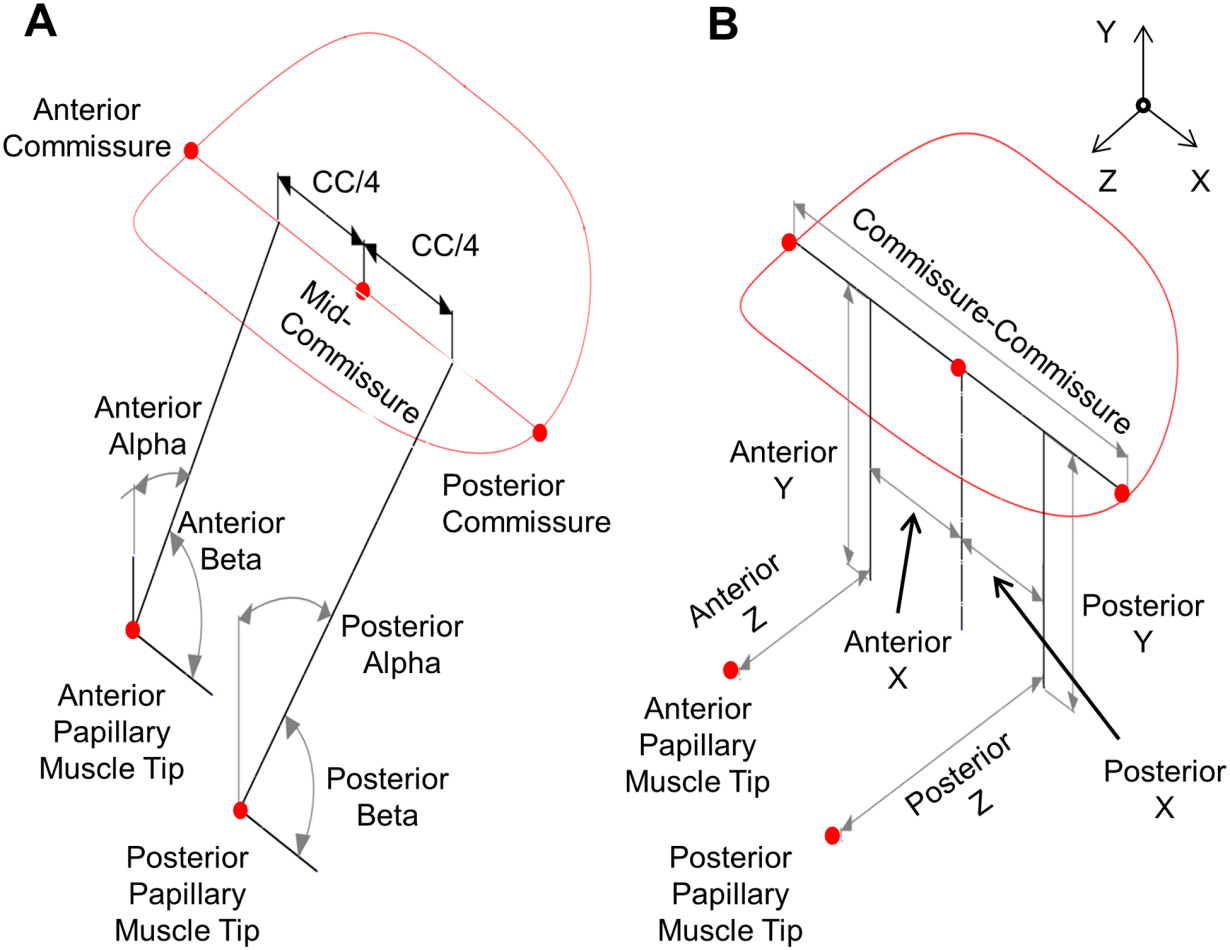
Papillary muscle holder A) Angular displacements and B) Cartesian coordinates.

The adjustment of the PM holder arms is completed with algorithms programmed into custom software. The commissure-PM tip and mid-anterior annulus-PM tip measurements uniquely define the location of each PM tip with respect to the annulus. For further definitions, please see S1 Appendix. Once the required measurements detailed earlier are found, they are entered into a Computer Aided Design (CAD) file as dimensions from reference points representing each PM tip to the respective annular features. When edited (please see S1 Appendix for details), these dimensions automatically recalculate a set of driven dimensions placed within a Cartesian coordinate frame with its origin at the mid-commissure point. The orientation of the muscle tips is controlled by maintaining the top surface of the muscle holder normal to a line projected from the PM location to the quarter-commissure point. In addition to Cartesian coordinates, altitude and azimuth angles, referred to respectively as alpha and beta, are automatically recalculated from each muscle tip to its corresponding quarter-commissure point. The automatically computed alpha and beta angles as well as Cartesian coordinates as well as the directions of the coordinate axes are presented and labeled in Fig 6.

**Fig 7:**
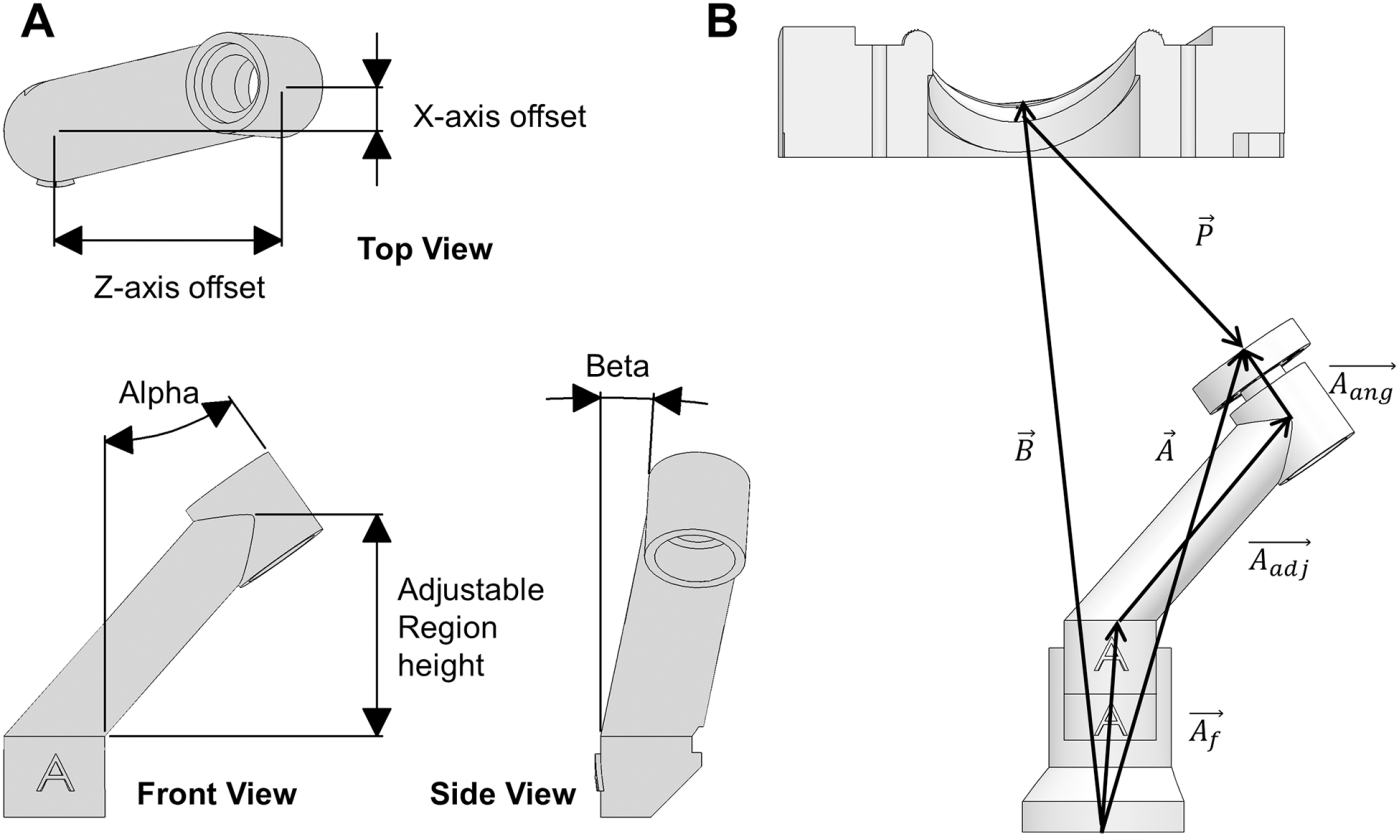
Papillary muscle holder A) Adjustment points and angles B) Calculation vector definitions: B: Mid-commissure to PM holder base, A: PM holder base to stitching ring, P: Commissure to PM tip position, A_f_: Fixed region, A_adj_: Adjustable region, A_ang_: Angled region.

The Cartesian and angular coordinates are manually entered into a custom software system to allow for alterations in case design. Through a set of intermediate calculations, the X and Z locations, the adjustable height, and the alpha and beta angles of the PM holder arms are computed and displayed. These values are then transferred to the CAD software in order to adjust the dimensions of each PM arm. The midpoint of the PM tip to the stitching ring is measured after installation of the fabric that wraps the PM for mounting, and this length is automatically subtracted from the PM to annulus distance and all angles are recalculated. The adjustment points for the PM holder arms are illustrated in Fig 7A, while the vectors used for calculating the dimensions of the PM holder arms are identified in Fig 7B. All vectors are taken to be three-dimensional, although shown only in two dimensions for clarity.

Following customization, the PM holder arms and annulus clamp are 3D printed using a stereolithographic printer (Objet30, Stratasys, Eden Prairie, Minnesota, USA) using VeroWhitePlus (Stratasys) model material. Printing and cleaning the mounting hardware takes approximately 5 hours, allowing for valves to be mounted relatively quickly following explantation. Stereolithography was used over extrusion based printing due to the superior resolution. All mounting hardware uses brass fittings for MRI safety and compatibility. The case and flanges were constructed from laser cut cast acrylic (Elipog Mini 24 30W, Epilog Laser, Golden, Colorado, USA).

### Mounting valves

**Fig 8:**
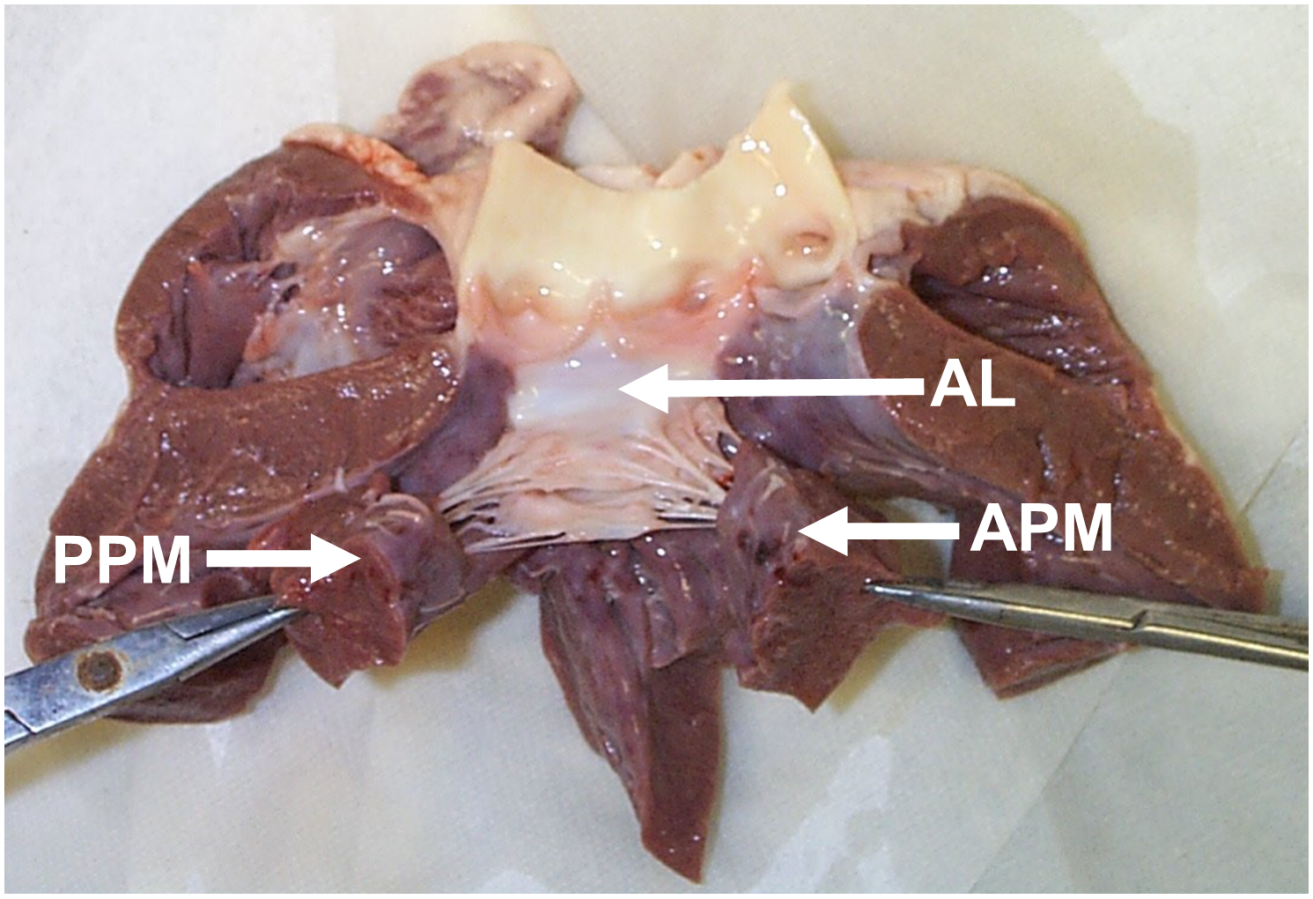
Explanting the mitral valve. AL: Anterior Leaflet, PPM: Posterior Papillary Muscle, APM: Anterior Papillary Muscle.

The MVs are explanted as following: The heart is positioned with the anterior portion pointing upward from the frontal plane. Using a straight scissor, the left auricle is removed. From a superior view, the left atrium descended into the annulus of the mitral valve, which is the visible “ring” defining the transition zone from myocardium to valvular tissue. The left thumb is inserted into the aorta as deeply as is possible without damaging the heart itself, creating a protective barrier to avoid potential damaging the MV leaflets, CT and PMs with incisions made through the heart tissue. Using a straight cutting scissor, the bottom metal prong was inserted into the aorta with the bottom resting on the thumb at the opposite side of the MV and the upper prong over the outside of the aorta. The heart is cut straight down the sagittal plane in the direction of the thumb to the apex of the heart. The heart opens to show the left and right atrioventricular junctions, and the MV appears (Fig 8). The PMs are removed from the LV myocardium, and the valve is detached from the left atrium and ventricle by cutting 5–10mm from the annulus into the atrial and ventricular myocardium. From this point, the valve is trimmed to leave approximately 10mm of PM tissue below the fibrous membranes and 5mm of tissue outside of the annulus around the circumference.

**Fig 9:**
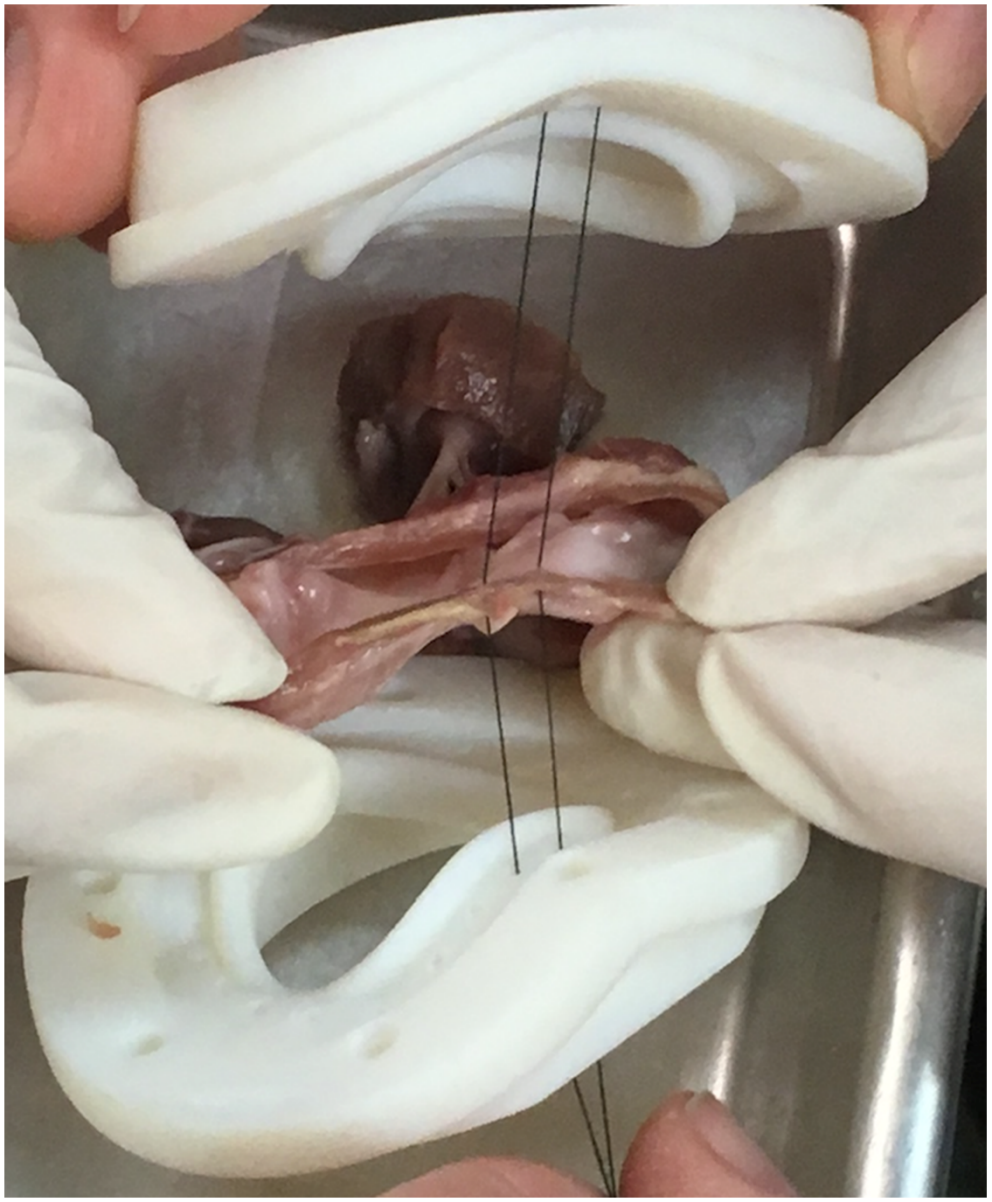
Mounting the mitral valve in the annulus clamp device. For demonstration purposes, the tissue guiding suture is shown without the annulus being wrapped in Dacron.

The tissue starting 1mm outside of the annulus is wrapped in a Dacron textile and secured to the tissue throughout the circumference surrounding the annulus. This is to ensure that the annulus can be mounted in both the diastolic and the systolic configuration without compromising the strength and integrity of the tissue. Fig 9 demonstrates how the valve is mounted on the annulus holder with suture. The surfaces clamping onto the annulus itself have contoured ripple edges facing towards the center of the valve to prevent slippage while avoiding destruction of the tissue integrity. Depending on valve size and annulus configuration, between four and eight sutures are placed 2–3 mm outside of the annulus for the purpose of keeping the valve straight and centered within the clamp halves and guide the valve into the correct position during mounting.

**Fig 10:**
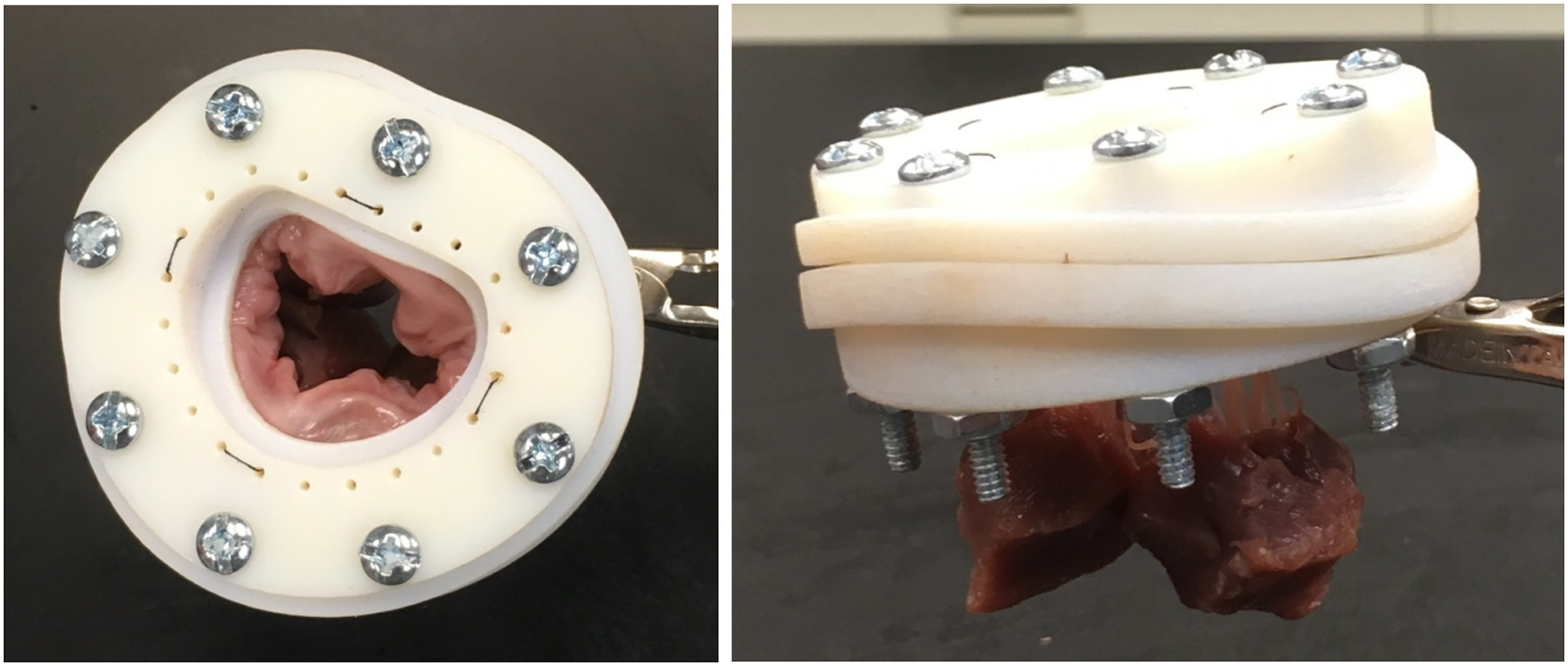
Annulus clamp with mounted valve viewed from top (right) and side view (left) showing papillary muscles. This valve is mounted without the PM holder tips for illustrative purposes.

Then the valve is mounted within the annulus clamp, secured by mechanical pressure exerted by the two rounded clamping surfaces as shown in Fig 2A. Fig 10 shows a valve mounted in the annulus clamp. The PMs are wrapped with fabric to facilitate attachment of the stitching rings [17], and this mount does not change between imaging configurations. The anterior and posterior PM holder arms are connected to the corresponding PM stitching ring and are then secured to the PM holder base. With the valve mounted, the assembly is inserted and secured within the case.

### Fluid pressure control system

**Fig 11:**
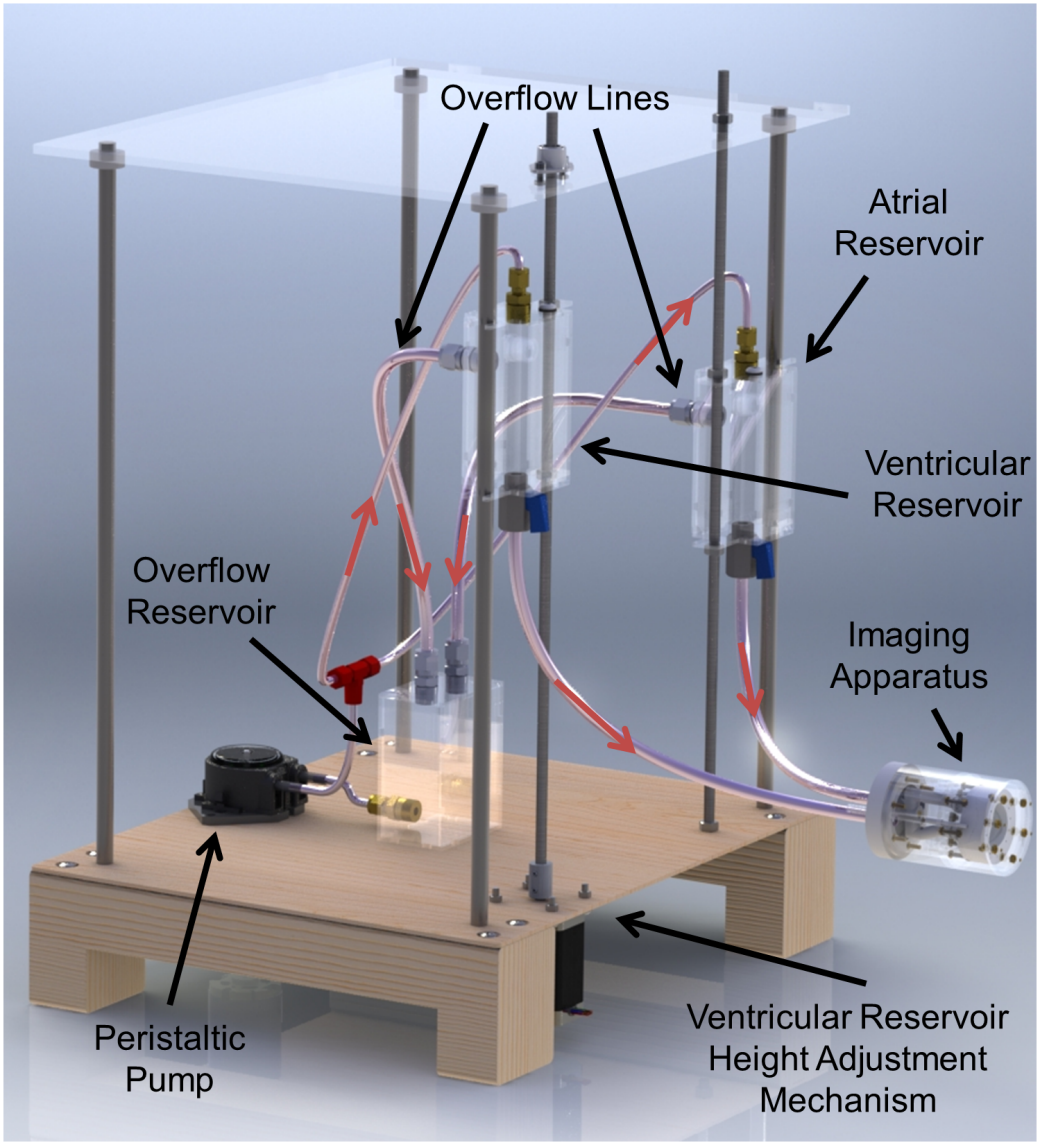
Imaging apparatus with fluid controls system for constant trans-mitral pressure generation.

The *in vitro* pressure across the valve membrane for simulating the systolic configuration is controlled experimentally with a hydrostatic constant pressure supply system applied to a fluid environment within the case. The fluid pressure on each side of the MV is kept constant by this control system utilizing differing fluid levels to control the relative hydrostatic pressure difference across the valve. Fluid heads for each side of the case are created by gravitational pressure from two fluid filled reservoirs of adjustable height difference. Each reservoir is equipped with spillovers that cause any additional fluid over a predetermined volume to spill into an overflow reservoir. Fluid is continually pumped from the overflow reservoir back into each of the atrial and ventricular reservoirs to ensure they remain constantly full up to the spillovers, thereby holding the pressure difference constant. At and above 10mmHg, the valve is fully closed and the collagen in the MV is fully recruited [4]. Beyond this pressure, the leaflets maintain stiffness without further deformation relevant to the imaging of the individual structures of the valve [18, 19]. Hence, it can be justified that 10mmHg is sufficient to image the valve in the fully closed configuration. The entire system is shown in Fig 11 with major components of the fluid controls system labeled. The direction of fluid flow is indicated by red arrows. For the unloaded diastolic configuration scan, the pressure across the membrane is held to zero, meaning that the supply tanks in Fig 11 are at the same vertical level.

The fluid environment used is Inland geminYe 04 Perfluorinated Polyether (PFPE) oil (Inland Vacuum, Churchville, New York). GeminYe oil is an excellent medium for this application since it lacks H1 nuclei, or protons, that would interfere with 7 Tesla MRI imaging. Additionally, the magnetic susceptibility of geminYe closely matches that of tissue. This prevents a sharp transition of susceptibility at the wetting interface that would cause distortion. The choice of 04 weight oil was made to provide an appropriate degree of neutral buoyancy to the tissue close to the density of blood. Viscous head losses within the plumbing of the fluid control system drove the necessity of relatively low viscosity oil. A peristaltic pump with a dampener is used so that the fluid is isolated and to prevent contamination. The system forms a closed loop with all oil contained within capped reservoirs. The pressure difference between chambers is finely adjusted by the use of a movable platform that raises and lowers the ventricular reservoir with respect to the atrial reservoir (see Fig 11).

### Imaging

The outside diameter of the case is slightly smaller than the 7T MRI system’s bore diameter such that the case can be inserted for imaging without the use of positioning guides. The fluid supply tubes for each chamber extend out the same side of the machine a safe distance before connecting to the fluid control system. Imaging was performed using a Bruker BioSpec 70/30AS MRI (Bruker, Billerica, MA) with Avance III console equipped with B-GA12 gradient insert (440 mT/m) and 72 mm birdcage volume RF coil. Images were acquired using fast low-angle shot (FLASH) pulse sequence with the following parameters: 640x640x382 matrix size, 80μm isotropic resolution, 3.7ms echo time, 30% echo shift, 20ms repetition time, 10 degree flip angle, and 16 averages. These parameters and the size of the valve resulted in imaging times of approximately 30 hours. Two or more datasets can be acquired, and if no motion or distortion between the images they can be averaged to increase the signal to noise ratio. This was not performed in this first pilot experiment.

## Results

**Fig 12:**
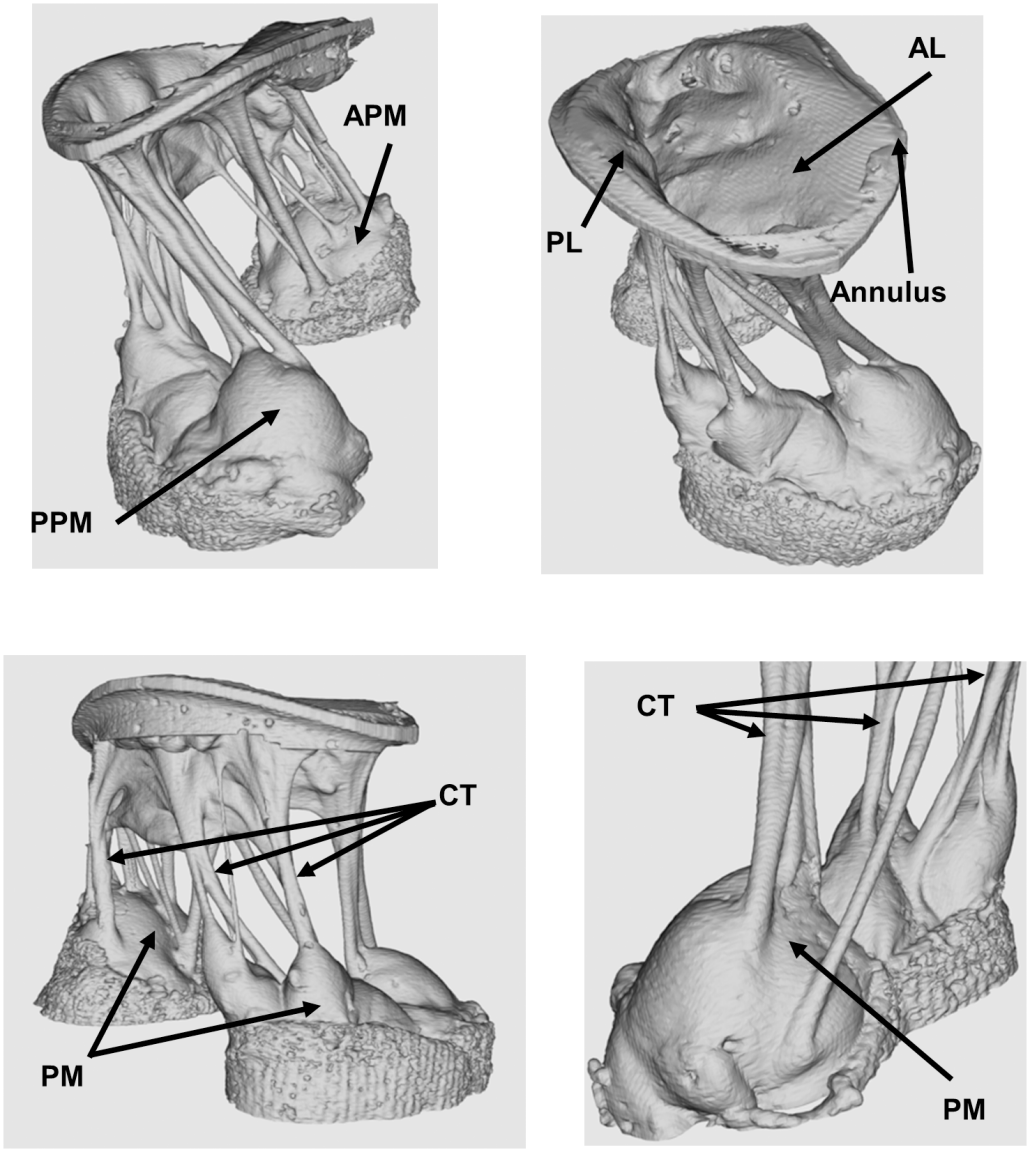
Example of the high level of details of the mitral valve structures obtainable by 7T MRI imaging. Ventricular and atrial views displayed, as well as enlarged views of the papillary muscles and chordae tendineae. The valve is 100% slack in a zero-pressure state. APM: Anterior Papillary Muscle, PPM: Posterior Papillary Muscle, CT: Chordae Tendineae, PM: Papillary Muscle, AL: Anterior Leaflet, PL: Posterior Leaflet.

Imaging was performed on an explanted porcine heart obtained from a local abattoir prior to TEE measurements for demonstrative purposes. Average dimensions for that size animal for this pilot study was obtained from literature [13]. Using the MV measurements, the mounting hardware was 3D printed and the valve is mounted for scanning. Fig 12 shows multiple angles of the 3D dataset.

Image processing to remove and mask artefacts from individual slices was performed using ImageJ version 1.51n. Artefacts in the dataset included portions of the valve mounting hardware and parts of the case. Following masking, the individual images were constructed into the 3D model using ImageJ’s 3D viewer plugin.

The images displayed in Fig 12 are from a valve in the unloaded state. Fluid is not flowing through the valve; hence it is not fully opened. The leaflets are relaxed and the CT are non-taut. The detailed emersion of the CT from the fibrous membrane of the CT is very clear, and the level of spatial information is of high detail. The CT are clearly separated from one another, and the insertion points, angles and geometry to the leaflets are as for the PMs very clearly illustrated.

**Fig 13:**
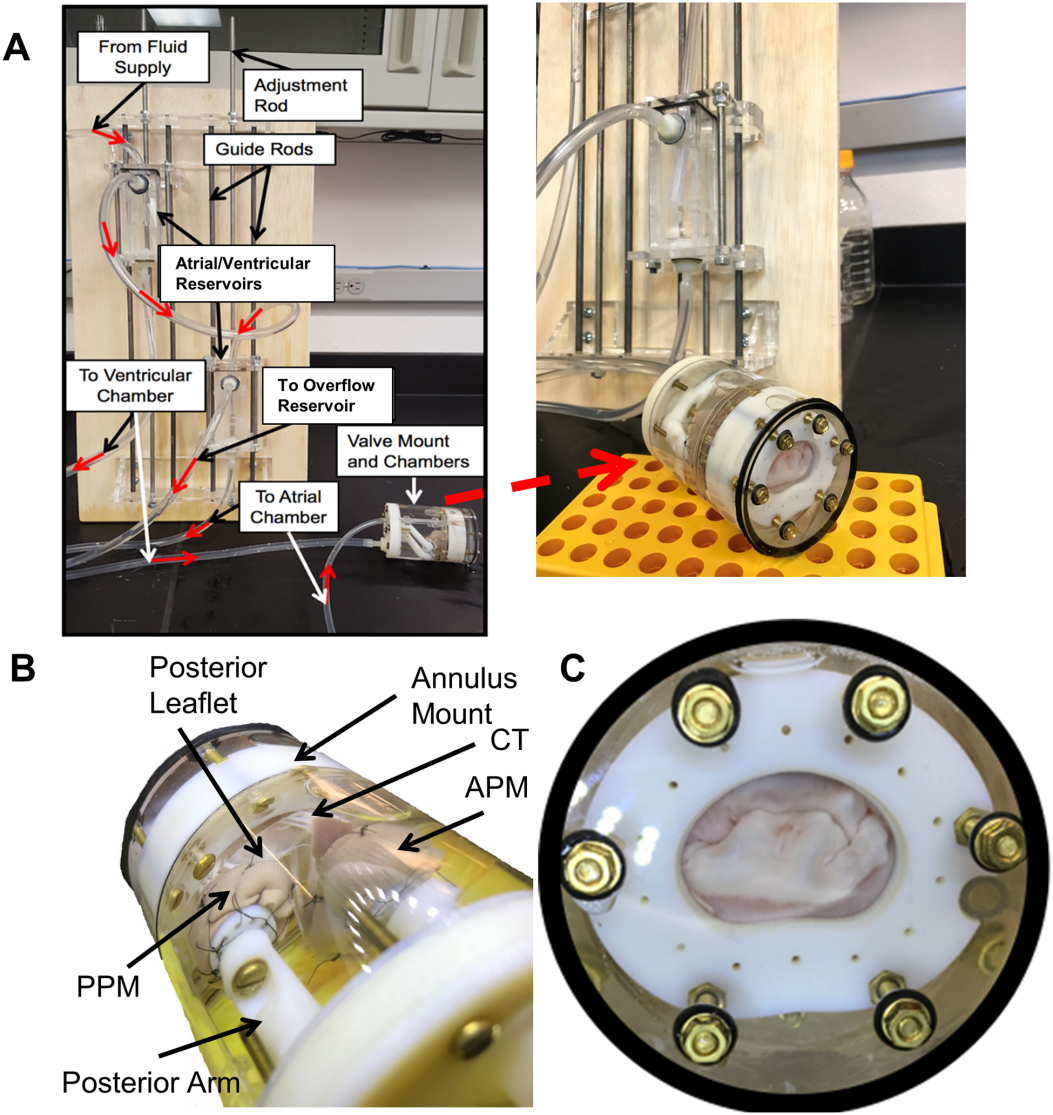
Pressure control system and valve pressurized in chamber. A) Control system as outlined in Fig 11, valve pressurized. B) Ventricular view of pressurized valve showing papillary muscle mounting. CT: Chordae Tendineae; APM: Anterior Papillary Muscle; PPM: Posterior Papillary Muscle C) Atrial view of pressurized valve showing a physiological leaflet coaptation pattern.

The trans-mitral pressure system was demonstrated to be able to control the 10mmHg pressure difference necessary for scanning the valve in the systolic configuration, see Fig 13. As no leaks were observed during a 30-minute test, it is reasonable to extrapolate that this system will keep leak tight for the duration of the scans.

## Discussion

The advantage of the new technique is multi-faceted. Although the resolution and quality of *in vitro* three-dimensional imaging techniques for the MV have improved greatly in recent years, the details of the valve in the unloaded natural state without any tissue structure alterations from compromising chemical treatments as well as subject specific physiological positioning of the anchoring points are still missing. In the current study, use of a fluid environment reduced the gravitational distortion the tissue experiences due to buoyancy, which is of particular importance in open valve or low pressure closed valve cases. In the diastolic configuration, the tissue is truly fully unloaded with zero pressure difference between the atrial and the ventricular chamber. The fluid keeps the valve tissue consistently moisturized during the imaging process, in both closed systolic and open diastolic configurations. Prior studies have shown no significant alteration to tissue immersed in PFPE oils, even for significant time periods [20]. As a result, this eliminates any issues with surface tension, tissue shrinkage, and CT bunching in the valve apparatus.

The newly developed system and methods are designed to use pre-mortem ultrasound based measurements of key MV dimensions as input to 3D print customized mounting hardware for each valve prior to imaging. This method allows for valves to be mounted and imaged in significantly more physiologically appropriate configurations, in order to mimic their own native environment. As individual valves can vary substantially in structure, the replication of native conditions minimizes non-physiologic leaflet strain, chordal force distribution, and coaptation pattern, all of which are directly impacted from annulus shape and PM position [4, 7, 9, 13, 21–28].

Additionally, the valve mounting hardware is designed such that the valve can be removed from the apparatus following imaging and inserted into a different apparatus (such as a force measurement chamber) without harming the integrity of the valve tissue. By replicating both diastolic and systolic conditions, the force exerted by the PMs through the chords and onto the leaflets can be quantified. The replication of a valve’s native shape using modular, customizable mounting hardware, coupled with the superior voxel resolution obtained through 7T MRI imaging and directly corresponding force measurements, stands to dramatically improve the validity of future work in the area of computational modeling.

It should be noted that direct validation of MV computational models requires high-fidelity three-dimensional data. These models of the heart and heart valves have the potential ability to simulate and predict the performance of medical devices and intervention techniques. This allows early feedback in the design process, ultimately reducing development time and improving patient outcome through personalized procedures. The overall accuracy and the reliability of such computational models however require high quality data in the unloaded state as well as data for the loaded state for geometric validation. Current models of the MV are based on *in vivo/vitro* imaging and bench-top measured or assumed material properties, without converging with actual biomechanical measurements. Including these measurements in a precise replica of individual valves would provide significant advantages to the computational models of the MV.

## Limitations

While the use of 7T MRI provides significant advantages as described, it also introduces several limitations. Due to the small bore diameter of the machine, the valve holding apparatus cannot exceed 72mm in diameter. This limits the maximum size of valves that can be accommodated with the apparatus, approximately 36mm commissure-commissure width. Certain PM configurations will not fit within the case, in terms of position as well as number. These restrictions limit the sizes and types of valves that can be used with the system, which has historically been an issue with in vitro modeling of atrioventricular valves. The acceptability of a given annulus geometry and PM configuration can (as indicated above) be verified prior to 3D printing by following the algorithm to generate the updated models and then checking the assembled apparatus model for interferences.

When mounting and assembling the system, tweezers are needed to grasp and position several components during assembly. As more experience is obtained on how to operate most efficiently within these tight spaces, future iterations of this design could see easier handling and dedicated tools for easier and faster mounting and disassembling. These limitations also impact the cleaning of the apparatus following imaging.

The imaging time is longer compared to high resolution 3D methods described previously, with for example microCT. This puts significant requirements of the steadiness of the system when imaging in the diastolic as well as the systolic phase. As shown in the resulting images, we have successfully managed to acquire images of a full valve. Future optimization to shorten the imaging time could be to acquire more scans with a lower resolution and less signal-to-noise ratio and post-process to obtain the same end-result.

The workflow described allows for superior replication of in vivo conditions of the mitral valve, requiring logistical foresight with several interdependent steps. If a valve fails towards the end of the process, the lost investment is considerable. This limitation is acceptable since it exists in the core nature of the project that will eventually seek to obtain detailed material properties of atrioventricular valves that can then be applied to a broad range of valves, when the imaging data is available.

## Conclusions and future work

The techniques and methodologies presented in this work were developed to mimic the environment of the MV apparatus in the highest level of physiologically appropriate conditions. Through pre-mortem based measurements, custom mounting hardware for the mitral annulus and PMs are printed to support the valve for high-resolution 3D imaging. Current computational models for predicting MV behavior rely upon material and geometrical assumptions that require validation, and the results from the work presented here will first of all create optimal geometry data. Future use of this system will be complimented by in vitro biomechanical measurements within the same valve, providing the optimal physiologically correct input for computational models of the MV. These models will enable more realistic simulation of repair and replacement strategies, as well as enable more accurate estimates about the long-term viability of the intervention.

## Supporting information

S1 Appendix Complete equation list and variable definitions for customizing PM holder. (DOCX)

## References

[1] Rabbah JP, Saikrishnan N, Yoganathan AP. A novel left heart simulator for the multi-modality characterization of native mitral valve geometry and fluid mechanics. Annals of biomedical engineering. 2013;41(2):305–15. doi: 10.1007/s10439-012-0651-z 22965640 10.1007/s10439-012-0651-z PMC3545111

[2] Bloodworth CHt, Pierce EL, Easley TF, Drach A, Khalighi AH, Toma M, et al Ex Vivo Methods for Informing Computational Models of the Mitral Valve. Annals of biomedical engineering. 2017;45(2):496–507. doi: 10.1007/s10439-016-1734-z 27699507 10.1007/s10439-016-1734-z PMC5300906

[3] Toma M, Bloodworth CHt, Einstein DR, Pierce EL, Cochran RP, Yoganathan AP, et al High-resolution subject-specific mitral valve imaging and modeling: experimental and computational methods. Biomech Model Mechanobiol. 2016;15(6):1619–30. doi: 10.1007/s10237-016-0786-1 27094182 10.1007/s10237-016-0786-1

[4] Jimenez JH, Liou SW, Padala M, He Z, Sacks M, Gorman RC, et al A saddle-shaped annulus reduces systolic strain on the central region of the mitral valve anterior leaflet. The Journal of thoracic and cardiovascular surgery. 2007;134(6):1562–8. doi: 10.1016/j.jtcvs.2007.08.037 18023684 10.1016/j.jtcvs.2007.08.037

[5] Jensen MO, Jensen H, Levine RA, Yoganathan AP, Andersen NT, Nygaard H, et al Saddle-shaped mitral valve annuloplasty rings improve leaflet coaptation geometry. The Journal of thoracic and cardiovascular surgery. 2011;142(3):697–703. doi: 10.1016/j.jtcvs.2011.01.022 21329946 10.1016/j.jtcvs.2011.01.022 PMC3224846

[6] Salgo IS, Gorman JH 3rd, Gorman RC, Jackson BM, Bowen FW, Plappert T, et al Effect of annular shape on leaflet curvature in reducing mitral leaflet stress. Circulation. 2002;106(6):711–7. 12163432 10.1161/01.cir.0000025426.39426.83

[7] Jimenez JH, Soerensen DD, He Z, Ritchie J, Yoganathan AP. Effects of papillary muscle position on chordal force distribution: an in-vitro study. The Journal of heart valve disease. 2005;14(3):295–302. 15974521

[8] He S, Weston MW, Lemmon J, Jensen M, Levine RA, Yoganathan AP. Geometric distribution of chordae tendineae: an important anatomic feature in mitral valve function. The Journal of heart valve disease. 2000;9(4):495–501; discussion 2–3. 10947041

[9] Cochran RP, Kunzelman KS. Effect of papillary muscle position on mitral valve function: relationship to homografts. The Annals of thoracic surgery. 1998;66(6 Suppl):S155–61. 9930439 10.1016/s0003-4975(98)01100-x

[10] Khalighi AH, Drach A, Bloodworth CHt, Pierce EL, Yoganathan AP, Gorman RC, et al Mitral Valve Chordae Tendineae: Topological and Geometrical Characterization. Annals of biomedical engineering. 2017;45(2):378–93. doi: 10.1007/s10439-016-1775-3 27995395 10.1007/s10439-016-1775-3 PMC7077931

[11] Toma M, Jensen MO, Einstein DR, Yoganathan AP, Cochran RP, Kunzelman KS. Fluid-Structure Interaction Analysis of Papillary Muscle Forces Using a Comprehensive Mitral Valve Model with 3D Chordal Structure. Annals of biomedical engineering. 2016;44(4):942–53. doi: 10.1007/s10439-015-1385-5 26183963 10.1007/s10439-015-1385-5 PMC4715994

[12] Jensen MO, Lemmon JD, Gessaghi VC, Conrad CP, Levine RA, Yoganathan AP. Harvested porcine mitral xenograft fixation: impact on fluid dynamic performance. The Journal of heart valve disease. 2001;10(1):111–24. 11206757

[13] Dagum P, Timek T, Green GR, Daughters GT, Liang D, Ingels NB Jr., et al Three-dimensional geometric comparison of partial and complete flexible mitral annuloplasty rings. The Journal of thoracic and cardiovascular surgery. 2001;122(4):665–73. doi: 10.1067/mtc.2001.116313 11581596 10.1067/mtc.2001.116313

[14] Levine RA, Handschumacher MD, Sanfilippo AJ, Hagege AA, Harrigan P, Marshall JE, et al Three-dimensional echocardiographic reconstruction of the mitral valve, with implications for the diagnosis of mitral valve prolapse. Circulation. 1989;80(3):589–98. 2766511 10.1161/01.cir.80.3.589

[15] Jensen MO, Jensen H, Smerup M, Levine RA, Yoganathan AP, Nygaard H, et al Saddle-shaped mitral valve annuloplasty rings experience lower forces compared with flat rings. Circulation. 2008;118(14 Suppl):S250–5. doi: 10.1161/CIRCULATIONAHA.107.746776 18824763 10.1161/CIRCULATIONAHA.107.746776

[16] Askov JB, Honge JL, Jensen MO, Nygaard H, Hasenkam JM, Nielsen SL. Significance of force transfer in mitral valve-left ventricular interaction: in vivo assessment. The Journal of thoracic and cardiovascular surgery. 2013;145(6):1635–41, 41.e1 doi: 10.1016/j.jtcvs.2012.07.062 22980066 10.1016/j.jtcvs.2012.07.062

[17] Jensen MO, Fontaine AA, Yoganathan AP. Improved in vitro quantification of the force exerted by the papillary muscle on the left ventricular wall: three-dimensional force vector measurement system. Annals of biomedical engineering. 2001;29(5):406–13. 11400721 10.1114/1.1366672

[18] Votta E, Caiani E, Veronesi F, Soncini M, Montevecchi FM, Redaelli A. Mitral valve finite-element modelling from ultrasound data: a pilot study for a new approach to understand mitral function and clinical scenarios. Philos Trans A Math Phys Eng Sci. 2008;366(1879):3411–34. doi: 10.1098/rsta.2008.0095 18603525 10.1098/rsta.2008.0095

[19] Kunzelman KS, Cochran RP. Stress/strain characteristics of porcine mitral valve tissue: parallel versus perpendicular collagen orientation. J Card Surg. 1992;7(1):71–8. 1554980 10.1111/j.1540-8191.1992.tb00777.x

[20] Hyare H, Powell C, Thornton J, Parkes H, Mancini L, Yousry T, et al Perfluoropolyethers in Magnetic Resonance Microscopy Effect on Quantitative Magnetic Resonance Imaging Measures and Histological Properties of Formalin-Fixed Brain Tissue. Proceedings of the International Society for Magnetic Resonance in Medicine 2008:16 p. 1719.

[21] He S, Jimenez J, He Z, Yoganathan AP. Mitral leaflet geometry perturbations with papillary muscle displacement and annular dilatation: an in-vitro study of ischemic mitral regurgitation. The Journal of heart valve disease. 2003;12(3):300–7. 12803328

[22] Rahmani A, Rasmussen AQ, Honge JL, Ostli B, Levine RA, Hagege A, et al Mitral valve mechanics following posterior leaflet patch augmentation. The Journal of heart valve disease. 2013;22(1):28–35. 23610985 PMC3644588

[23] Jimenez JH, Soerensen DD, He Z, He S, Yoganathan AP. Effects of a saddle shaped annulus on mitral valve function and chordal force distribution: an in vitro study. Annals of biomedical engineering. 2003;31(10):1171–81. 14649491 10.1114/1.1616929

[24] Lai DT, Tibayan FA, Timek TA, Liang D, Daughters GT, Ingels NB Jr., et al Three-dimensional in-vivo dimensions of 'He's triangle' during acute left ventricular ischemia. JHeart Valve Dis. 2001;10(6):767–73. 11767184

[25] Timek TA, Lai DT, Liang D, Tibayan F, Langer F, Rodriguez F, et al Effects of paracommissural septal-lateral annular cinching on acute ischemic mitral regurgitation. Circulation. 2004;110(11 Suppl 1):II79–84. doi: 10.1161/01.CIR.0000138975.05902.a5 15364843 10.1161/01.CIR.0000138975.05902.a5

[26] Timek TA, Lai DT, Tibayan F, Liang D, Daughters GT, Dagum P, et al Septal-lateral annular cinching abolishes acute ischemic mitral regurgitation. The Journal of thoracic and cardiovascular surgery. 2002;123(5):881–8. 12019372 10.1067/mtc.2002.122296

[27] Jimenez JH, Soerensen DD, He Z, Ritchie J, Yoganathan AP. Mitral valve function and chordal force distribution using a flexible annulus model: an in vitro study. AnnBiomedEng. 2005;33(5):557–66. 10.1007/s10439-005-1512-9 15981857

[28] Padala M, Hutchison RA, Croft LR, Jimenez JH, Gorman RC, Gorman JH 3rd, et al Saddle shape of the mitral annulus reduces systolic strains on the P2 segment of the posterior mitral leaflet. The Annals of thoracic surgery. 2009;88(5):1499–504. doi: 10.1016/j.athoracsur.2009.06.042 19853100 10.1016/j.athoracsur.2009.06.042 PMC3021783

