# Supplementary material for: High resolution imaging of the mitral valve in the natural state with 7 Tesla MRI
Source: PLoS One. 2017 Aug 30;12(8):e0184042. doi: 10.1371/journal.pone.0184042 (PMC5576658; doi:10.1371/journal.pone.0184042)
Supplement: S1 Appendix — (DOCX) [file pone.0184042.s001.docx]

**S1 Appendix. Complete equation list and explanation of variables**

**Explanation of Variables**

**APMT:** Anterior papillary muscle tip location

**PPMT:** Posterior papillary muscle tip location

**CP:** Commissure plane

**BT:** Top of papillary muscle holder base

**PMW:** Papillary muscle holder base width

**PMAL:** Papillary muscle holder arm angled region length

**PMFL:** Papillary muscle holder arm fixed portion length

**δ:** Papillary muscle holder base centerline to commissure-commissure line

**PMT:** Trimmed papillary muscle thickness

**X_a_, Y_a_, Z_a_:** Anterior papillary muscle tip X, Y, and Z Cartesian coordinates

**X_p_, Y_p_, Z_p_:** Posterior papillary muscle tip X, Y, and Z Cartesian coordinates relative to annulus mid-commissure point

**ΔX_a_, ΔY_a_, ΔZ_a_:** Anterior X, Y, and Z Cartesian coordinate modifications due to papillary muscle holder top angular displacement

**ΔX_p_, ΔY_p_, ΔZ_p_:** Posterior X, Y, and Z Cartesian coordinate modifications due to papillary muscle holder top angular displacement

**α_a_, β_a_:** Anterior papillary muscle tip angular displacements

**α_p_, β_p_:** Posterior papillary muscle tip angular displacements

**PMX_a_, PMZ_a_:** Anterior papillary muscle holder arm X and Z offsets

**PMX_p_, PMZ_p_:** Posterior papillary muscle holder arm X and Z offsets

**PML_a_:** Anterior papillary muscle holder arm adjustable length

**PML_p_:** Posterior papillary muscle holder arm adjustable length

**A_a_, B_a_:** Anterior alpha and beta plane angular offsets

**A_p_, B_p_:** Posterior alpha and beta plane angular offsets

**Papillary muscle holder calculations**

(APMT - BT)|_apical_ = (CP - BT)|_apical_ - Y_a_ (1)

(PPMT - BT)|_apical_ = (CP – BT)|_apical_ - Y_p_ (2)

ΔX_a_ = PMAL*cos(β_a_) - PMT* cos(β_a_) (3)

ΔX_p_ = PMAL*cos(β_p_) - PMT* cos(β_p_) (4)

ΔY_a_ = PMAL*sin(β_a_)*cos(α_a_) + PMT* sin(β_a_)*cos(α_a_) (5)

ΔY_p_ = PMAL*sin(β_b_)*cos(α_p_) + PMT* sin(β_b_)*cos(α_p_) (6)

ΔZ_a_ = PMAL*sin(α_a_) + PMT* sin(α_a_) (7)

ΔZ_p_ = PMAL*sin(α_p_) + PMT* sin(α_p_) (8)

PMX_a_ (Inward) =PMW - X_a_ - ΔX_a_ (9)

PMX_p_ (Inward) = PMW - X_p_ - ΔX_p_ (10)

PMZ_a_ (Toward Posterior) = Z_a_ + ΔZ_a_ – δ (11)

PMZ_p_ (Toward Posterior) = Z_p_ + ΔZ_p_ – δ (12)

PML_a_ = (APMT - BT)|_apical_ - ΔY_a_ – PMFL (13)

PML_p_ = (PPMT - BT)|_apical_ - ΔY_p_ – PMFL (14)

A_a_ = α_a_  (15)

A_p_ = α_p_ (16)

B_a_ = 90 - β_a_ (17)

B_p_ = 90 – β_p_ (18)

**Determining Cartesian Coordinates**

Echocardiographic measurements are used to determine the locations of both papillary muscle tips with respect to the annulus. For convenience, the locations of the muscle tips are described by a set of Cartesian coordinates with the origin located at the mid-commissure point. Analytical determination of the Cartesian coordinates based upon echo measurements can be accomplished by finding the intersection point of three spheres, a process called trilateration. These spheres, whose centers are located at each commissure and mid-anterior annulus, have radii that are given by the echo measurements from the corresponding feature to the muscle tips. Finding an analytical solution for the intersection points is nontrivial and falls outside the scope of this paper. The work presented utilizes Solidworks to determine the Cartesian coordinates through a set of driven dimensions that automatically recalculate upon changing any of the driven dimensions which represent measured values. In the future the authors would like to develop an algorithm to determine these values without the use of Solidworks.
